# Expression shifts of floral symmetry genes correlate to flower actinomorphy in East Asia endemic *Conandron ramondioides* (Gesneriaceae)

**DOI:** 10.1186/s40529-018-0242-x

**Published:** 2018-10-29

**Authors:** Kuan-Ting Hsin, Chun-Neng Wang

**Affiliations:** 1 Institute of Ecology and Evolutionary Biology, National Taiwan University, Taipei, Taiwan; 2 Department of Life Science, National Taiwan University, Taipei, Taiwan

**Keywords:** Peloria, Reversal, Zygomorphy, *CYCLOIDEA*, *DIVARICATA*, *RADIALIS*, Bilateral symmetry

## Abstract

**Background:**

Bilateral symmetry flower (zygomorphy) is the ancestral state for Gesneriaceae species. Yet independent reversions to actinomorphy have been parallelly evolved in several lineages. *Conandron ramondioides* is a natural radially symmetrical species survived in dense shade mountainous habitats where specialist pollinators are scarce. Whether the mutations in floral symmetry genes such as *CYC*, *RAD* and *DIV* genes, or their expression pattern shifts contribute to the reversion to actinomorphy in *C. ramondioides* thus facilitating shifts to generalist pollinators remain to be investigated. To address this, we isolated putative orthologues of these genes and relate their expressions to developmental stages of flower actinomorphy.

**Results:**

Tissue specific RT-PCR found no dorsal identity genes *CrCYCs* and *CrRADs* expression in petal and stamen whorls, while the ventral identity gene *CrDIV* was expressed in all petals. Thus, ventralized actinomorphy is evolved in *C. ramondioides*. However, *CrCYCs* still persists their expression in sepal whorl. This is congruent with previous findings that *CYC* expression in sepals is an ancestral state common to both actinomorphic and zygomorphic core Eudicot species.

**Conclusions:**

The loss of dorsal identity genes *CrCYCs* and *CrRADs* expression in petal and stamen whorl without mutating these genes specifies that a novel regulation change, possibly on cis-elements of these genes, has evolved to switch zygomorphy to actinomorphy.

**Electronic supplementary material:**

The online version of this article (10.1186/s40529-018-0242-x) contains supplementary material, which is available to authorized users.

## Background

Evolutionary reversal to actinomorphy (flower radial symmetry) from zygomorphy (flower bilateral symmetry) have occurred multiple times independently across flowering plant diversification (Hileman 2014). Although zygomorphy enhances pollination specialization, these reversals may have evolved in a benefit of increased pollinator generalization when pollinators are scarce or in harsh conditions (Cronk and Möller 1997; Donoghue et al. 1998). The frequent transitions of floral symmetry reversals along many flowering plant lineages imply that a similar or modified developmental program has been independently recruited for floral symmetry transition (Zhang et al. 2013; Hileman 2014).

Gesneriaceae species are predominantly bilateral flower symmetry (zygomorphy) and exhibit a great diversity of floral forms (Endress 1999, 2001; Weber 2004). Their flowers have been adaptively evolved with bee, fly, moth, birds and even bat into a variety of pollination syndromes (Harrison et al. 1999; Perret et al. 2007). However, there are also certain lineages evolved with flower reversion to actinomorphy. There are at least five and four independent reversals to actinomorphy events occurred in old world and new world Gesneriaceae lineages, respectively (Wang et al. 2010; Smith et al. 2004; Clark et al. 2011). This frequency of reversals to actinomorphy in Gesneriaceae is the highest among Lamiales, probably because it is the basal most lineage of Lamiales which is just derived from the ourgroup actinomorphic Oleaceae species (Endress 1999). The repeated reversals to actinomorphy in Gesneriaceae species also implied there are perhaps similar yet modified developmental programs repeatedly recruited among these reversals.

It has been argued that the reversals to actinomorphy may contain selection disadvantage by losing its specific pollinators (Cronk and Möller 1997). However, in the aforementioned examples, the actinomorphic floral forms caused by reversals in Gesneriaceae species are usually compromised by pollinator shifts and pollination strategies switched from nectar to pollen rewards (Weber 2004). The reversal to actinomorphy with corolla tube fully opened to attract every kinds of general pollinators may be selected for when only few pollinators are available (Cronk and Möller 1997). For example, the European relict actinomorphic species in Pyrenees, *Ramonda myconi*, has been inferred that reversion to actinomorphy by opening of the corolla tube provides adaptive advantage to attract more available general pollinators in harsh alpine habitat (Cronk and Möller 1997; Picó et al. 2002). Additionally, its pollination syndrome has shifted from nectar reward which is usually favored by specific pollinators to pollen reward allowing general pollinators to visit (Weber 2004; Wang et al. 2010).

*Conandron ramondioides* is another relic and paleoendemic genus in East Asia which is also apparently evolved as an actinomorphic reversal from zygomorphic ancestor (Kokubugata and Peng 2004; Wang et al. 2010; Xiao et al. 2012). Like *R. myconi*, its corolla tube is lost and five petals are equally large with five stamens fully developed. These indicate they are natural peloria with complete actinomorphy. Nectary glands of *C. ramondioides* are lost. Also, its stamens are dehisced by apical pores and pollens are powdery suggest they are pollinated by pollen-collecting bees (Wang, pers. obs.). *Conandron ramondioides* can only survive in limestone cliff, often in deep shade forest, where pollinators are scarce. Taken together, the reversal to actinomorphy in *C. ramondioides* could prevent it from relying only on specific pollinators because insect activity is relative low under this habitat.

The establishment of flower zygomorphy requires *CYCLOIDEA* (*CYC*) gene specifically expressed in dorsal side of the flower to promote growth difference between dorsal and ventral petals and retarding dorsal stamen (Luo et al. 1996, 1999; Corley et al. 2005). Thus the reversal to actinomorphy in *C. ramondioides* could probably results from loss of *CYC* function or a *CYC* expression shifts in flower bud. In *Antirrhinum majus*, mutation of *CYC* and its paralog *DICH* can result in complete actinomorphy with all petals equal in size resembling the ventral one and no retardation on stamens. *CYC* in *C. ramondioides* may also be mutated thus becoming actinomorphy. However, *CYC* and *DICH*’s effect is through activating a downstream MYB family gene *RADIALIS* (*RAD*) at dorsal side, whose encoded protein restricts another MYB-like protein *DIVARICATA* (*DIV*) to the ventral region (Corley et al. 2005; Costa et al. 2005; Raimundo et al. 2013). Thus in addition to *CYC*, fully functional *RAD* is also needed to develop flower zygomorphy. In *cyc dich* double mutant, *RAD* could not be activated to restrict ventral identity gene *DIV* to ventral region resulting in all petals resembling to ventral petal of wild type. This type of actinomorphic reversal due to mutation of *CYC* and/or its downstream *RAD* therefore is often called abaxialized (ventralized) effect (Cronk 2006; Zhang et al. 2013; Hileman 2014; Spencer and Kim 2017).

In contrast to ventralization, actinomorphy can be established through expanded expression of *CYC* and its homologues in petal whorl, an adaxialized (dorsalized) effect. In actinomorphic species such as *Cadia* of legumes and certain Malpighiaceae species, the expression of *CYC* extended from dorsal regions to lateral and ventral regions of the corolla (Citerne et al. 2006; Zhang et al. 2013). It thus appears that both the loss of *CYC*-like gene expression (ventralization) and the expansion (dorsalization) of *CYC*-like gene expression are two major mechanisms in creating flower actinomorphic reversal in angiosperm.

In Gesneriaceae, reversal to actinomorphy through both ventralization or dorsalization were reported (e.g. *Bournea leiophylla*, *Tengia scopulorum and Saintpaulia ionantha*) (Zhou et al. 2008; Pang et al. 2010; Hsu et al. 2018). In *B. leiophylla*, the *BlCYC1* and *BlRAD* genes were transiently expressed in floral meristem initiation stage and then quickly vanished at latter developmental stages. The loss of *CYC* expression at later stages correlates with the fact that all petals resembling ventral ones, demonstrating a ventralization form of reversal (Zhou et al. 2008). Unlike *B. leiophylla*, *CYC*-like expression in *T. scopulorum* has ubiquitously expressed in all petals across dorsoventral axis, a dorsalized form of actinomorphic reversal (Pang et al. 2010). Partial *CYC* sequences have been isolated from both *C. ramondioides* and *R. myconi* but no apparent SNP mutations result in premature stop codon in their coding regions (Xiao and Wang 2007; Picó et al. 2002). These imply *CYC* in *C. ramondioides* may still function. It would therefore essential to investigate whether expression patterns of *CYC* together with downstream *RAD* and *DIV* shifts, which may correlate and explain the developmental switch of actinomorphic reversal in *C. ramondioides*.

To examine possible roles of floral symmetry genes (homologues of *CYC*, *RAD* and *DIV*) involving in establishment of actinomorphic in *C. ramondioides*, we examined their expression patterns along floral developmental stages and separated floral organs. In order to ascertain the developmental process of actinomorphy in *C. ramondioides*, we also observed the bud development using scanning electron microscope. From these results, we hope to find whether there is a correlation between shifts of expression patterns among these floral symmetry genes and corresponded floral symmetry transition.

## Materials and methods

### Floral development in *C. ramondioides*

Floral buds of *C. ramondioides* were collected from top of inflorescence containing developing floral organ primordia for SEM examination. The materials were fixed in FAA overnight then transfer to 70% EtOH for preservation. Fixed materials were pre-dissected under stereo microscope S8APO (Leica) then dehydrated through an ethanol series (85%, 95%, and 100% twice) with each step for 20 min. After dehydration in 100% ethanol, materials were dehydrated through ascending gradients of acetone, dried with molecular sieve, and finally dried in a critical point dryer (Hitachi E101). Dried samples were mounted on aluminum stubs and then coated with gold–palladium using Hitachi E1011I sputter. Specimens were viewed using FEI SEM at working distance at 10 mm, and operating at 15 kV. Stages of flower development were summarized in Additional file 1: Table S1.

### Isolation and characterization of *CYC*, *RAD* and *DIV* homologues

*CYC*, *RAD* and *DIV* homologues were isolated from *C. ramondioides* total genomic DNA by using degenerate primer pairs. We used either previously published primers which have been claimed able to amplify all possible gene copies, or newly designed primers located at conserve domain of each gene to isolate these floral symmetry gene copies. For example, for isolating *CYC* homologues from *C. ramondioides*, a pair of Geseneriaceae specific primers, GcycFS and GcycR, were used for amplifying *CYC* homologues (Möller et al. 1999). For *RAD*, primers were design from conserved MYB domain with all available GenBank sequences from Gesneriaceae species and *Antirrhinum*. Similarly, *DIV* primers were design from conserved R2 and R3 domain. To isolate *CYC* homologues from *C. ramondioides*, The PCR products were then cloned into pGEM-T easy vector system (Promega, USA) and 8 clones were sequenced for checking numbers of *CYC* homologues of *C. ramondioides*. Then, to isolate *RAD* homologues of *C. ramondioides*, degenerate primer pair QAL-F (5′-RTTRGCRGTKTAYGACA-3′) and FPN-R (5′-TTYCCYAAYTACWGGACCA-3′) locating at conserve MYB domain and conserve 3′ end were designed according to available *RAD* homologues of other Gesneriaceae species. Last, to isolate *DIV* homologues of *C. ramondioides*, degenerate primer pair DIV-F MEI (5′-ATGGAGATTTTRDCMCCAAGTT-3′) and DIV-YGK-R1 (5′-CTCCARTCYCCYTTYCCATA-3′) locating at R2 end and R3 domain were designed based on available Gesneriaceae sequences and *DIV* form *A. majus* respectively. Both *RAD* and *DIV* homologue PCR products were cloned and sequenced (8 clones for examining *RAD* homologue; 7 clones for examining *DIV* homologue) following the same process as previously mentioned above. To extend into the 5′ and 3′ unknown sequence region of amplified *RAD* and *DIV* partial sequence above, rapid amplification of cDNA ends (5′- and 3′-RACE, SMART RACE cDNA amplification kit, Clontech) technique is applied for obtaining full length cDNA according to manufacturer suggestion. To investigate the homology of isolated *CYC*, *RAD* and *DIV* of *C. ramondioides*, we aligned full length sequences of them with their genbank available homologs from subfamily Didymocarpoideae where it belongs to and those from closely related model species *Antirrhinum majus* (Scrophulariaceae) (Luo et al. 1996; Almeida et al. 1997; Zhou et al. 2008; Yang et al. 2012).

### Phylogenetic analysis of isolated *CYC*, *RAD* and *DIV* homologues in *C. ramondioides*

To check the homology of these isolated *CYC*, *RAD* and *DIV* genes from *C. ramondioides*, available NCBI homologues of Gesneriaceae species, *Antirrhinum majus* and *Arabidopsis thaliana* were downloaded and used for reconstructing their phylogenies respectively. Sequences used to reconstruct phylogeny were listed in Additional file 2: Table S2. Nucleotide sequences were first translated into amino acid and aligned using default settings in CLUSTALX (Thompson et al. 1997) with major domains specified, then manually aligned afterward. We apply both neighbor joining (NJ) and maximum likelihood (ML) algorithm for testing the robustness of reconstructed *CYC*, *RAD* and *DIV* phylogeny. The NJ tree of each gene dataset was reconstructed using MEGA 6 (Tamura et al. 2013). For ML tree, the web interface PhyML 3.0 was applied (Guindon et al. 2010). Best-fit nucleotide substitution model of each dataset was evaluated by smart model selection (SMS) which is implementing in PhyML 3.0 (Lefort et al. 2017). For *CYC*-like gene dataset, the best-fit model is HKY + G model. For *RAD*, TN93 + G model is suggested and GTR + I + G model for *DIV.*

### Locus-specific RT-PCR

**Fig. 1:**
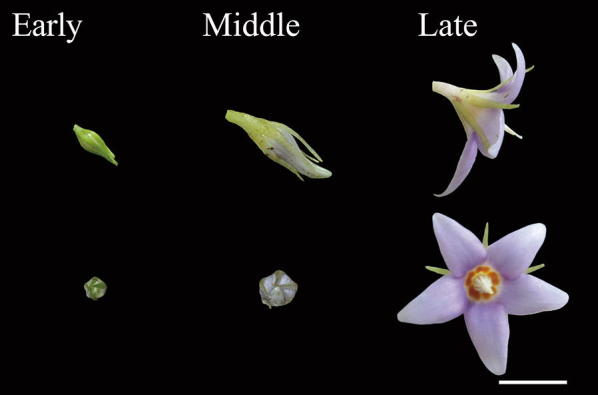
The flower of *C. ramondioides* from early to late stage. Early, middle and late stage corresponding to Stage 10, 13, 15 in Additional file 1: Table S1. Actinomorphy was observed in late stage *C. ramondioides* flower. Scale bar represented 1 cm

Flower buds were categorized into three stages (Fig. 1): early stage (Stage 10, 2–4 mm in diameter, sepal longer than petal), middle stage (Stage 13, 5–7 mm in diameter, petal longer than sepal), and late stage (Stage 15, anthesis) (see Additional file 1: Table S1). They were collected, in the field through fixing in RNAlater (Ambion, Life technologies, USA), or freshly collected from individuals grown in the greenhouse. Next, to detect expression locations of *CYC*, *RAD* and *DIV* homologues on petals, a single petal was dissected from flower buds at early developmental stage. Because RNA yield may be low in single petal, pooled sepals, petals, stamen and gynoecium were dissected from flower buds at early stage to confirm the expression locations of *CYC*, *RAD* and *DIV* homologues in *C. ramondioides*. Total RNA of floral buds and dissected floral organs were extracted following TRIzol^®^ Reagent (Invitrogen, USA) protocol. Single-strand cDNA (20 ng/μl) were reverse transcribed from total RNA of these samples by SuperscriptIV Reverse Transcriptase (Invitrogen). Gene specific primer pairs were used to examine each candidate gene’s expression level: *CrCYC1C* (forward: 5′-AGACATGCTTTCTGGCCACT-3′, Reverse: 5′-CTTCTTCGCCTTCTGAATGC-3′), *CrCYC1D* (Forward: 5′-CAGGTGCAGATTCGATGAGA-3′, Reverse: 5′-GTTCCATTGCAGTCTCCCAT-3′), *CrCYC2* (Forward: 5′-TCTTGCTTCATCAGCACCAC-3′, Reverse: 5′-GTGATGCCCCTACTTGCACT-3′), *CrRAD1* (Forward: 5′-ATGGCCTCAAGTTCCTTGACCGCT, Reverse: 5′-GTGGTCCAGTAGTTGGGAAATGGT), *CrRAD2* (Forward: 5′-GCAATGAGCTCCATGTCTAGTGGT, Reverse: 5′-CCCACTTCGATATTCTTCACATCCTCC-3′) and *CrDIV* (Forward: 5′-CGATCGATGGCAAAGAGTGG-3′, Reverse: 5′-TACTCGAACACCAACCCAGG-3′). The RT-PCR mixture contains 12 μl of Ampliqon master mix Red III (Denmark), 6.25 mM of each primer, 9.5 μl ddH_2_O and 1 μl first-strand cDNA. The PCR condition used for amplifying *CrCYC1C*, *CrCYC1D* and *CrCYC2* genes were 94 °C for 3 min followed by 35 cycles of 94 °C for 30 s, 53 °C for 40 s, 72 °C for 20 s and a 2 min final extension at 72 °C. To amplify higher Tm *CrRAD1* and *CrRAD2* genes, the program is 94 °C for 3 min followed by 35 cycles of 94 °C for 30 s, 57 °C for 40 s, 72 °C for 20 s and a 2 min final extension at 72 °C. For *CrDIV* gene, the program is 94 °C for 3 min followed by 35 cycles of 94 °C for 30 s, 53 °C for 40 s, 72 °C for 20 s and a 2 min final extension at 72 °C. *Cr18S* was used as positive control. Two biological replicates were carried out to validate the reproducibility of the results (see Additional file 3: Figure S1).

## Results

### Floral development

**Fig. 2:**
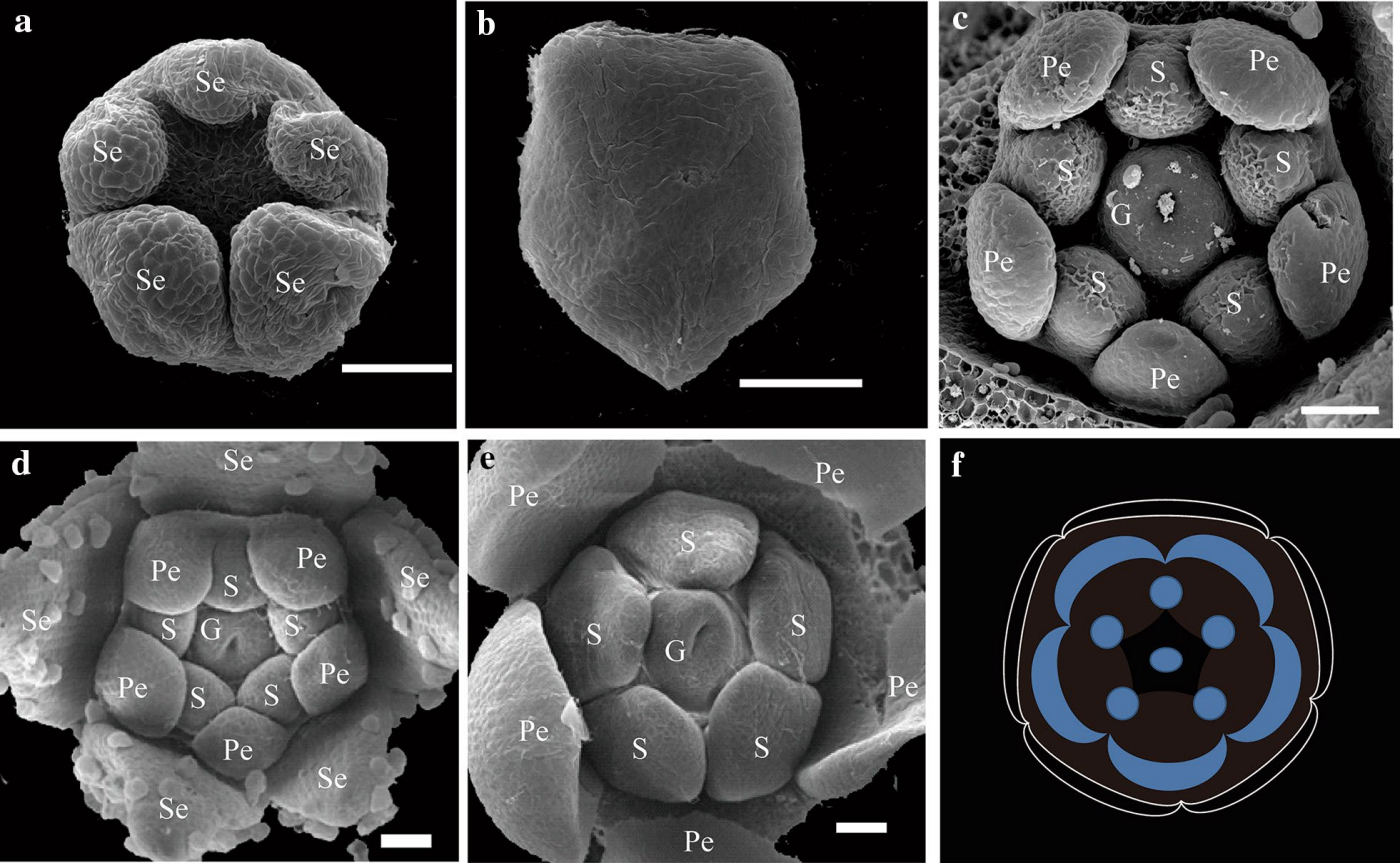
The SEM photos of morphological development process of *Conandron ramondioides* flowers. Zygomorphy was observed at Stage 3 of sepal whorl. Definition of developmental stages of *C. ramondioides* based on (Harrison et al. 1999). **a** Stage 3, the sepals form as bulges at the points of the pentagon. **b** Stage 4, the sepals grow, while the floral meristem remains undifferentiated. **c** Stage 6, the corolla and androecium grow, and the gynoecium initiates. **d** Stage 7A, petal growth. **e** Stage 7B, androecium and gynoecium development. **f** Floral diagram of *C. ramondioides*. *Se* Sepals, *Pe* petals, *S* stamen, *G* gynoecium. Scale bar represents 50 μm

Development of flower in *C. ramondioides* can be divided into 15 milestone stages (Additional file 1: Table S1). From Stage 3 SEM picture (Fig. 2a), all five sepals were already initiated but dorsal and lateral sepals were slightly smaller than ventral ones (a residual zygomorphy). During Stage 7 (sepal removed), petals and stamens appeared to be equaled in size when initiated (Fig. 2c). This is more evident during Stage 7A when gynoecium started to emerged, in which all petals and stamens were grown in equal size (Fig. 2d). Petals and stamens continued to grow in equal rate thus all 5 petals and stamens are the same size toward anthesis (Fig. 2e). The floral diagram of fully developed *C. ramondioides* thus can be drawn as Fig. 2f, showing complete actinomorphy of *C. ramondioides* flower at anthesis.

### Characterization and phylogeny of *CrCYC*, *CrRAD* and *CrDIV* genes

**Fig. 3:**
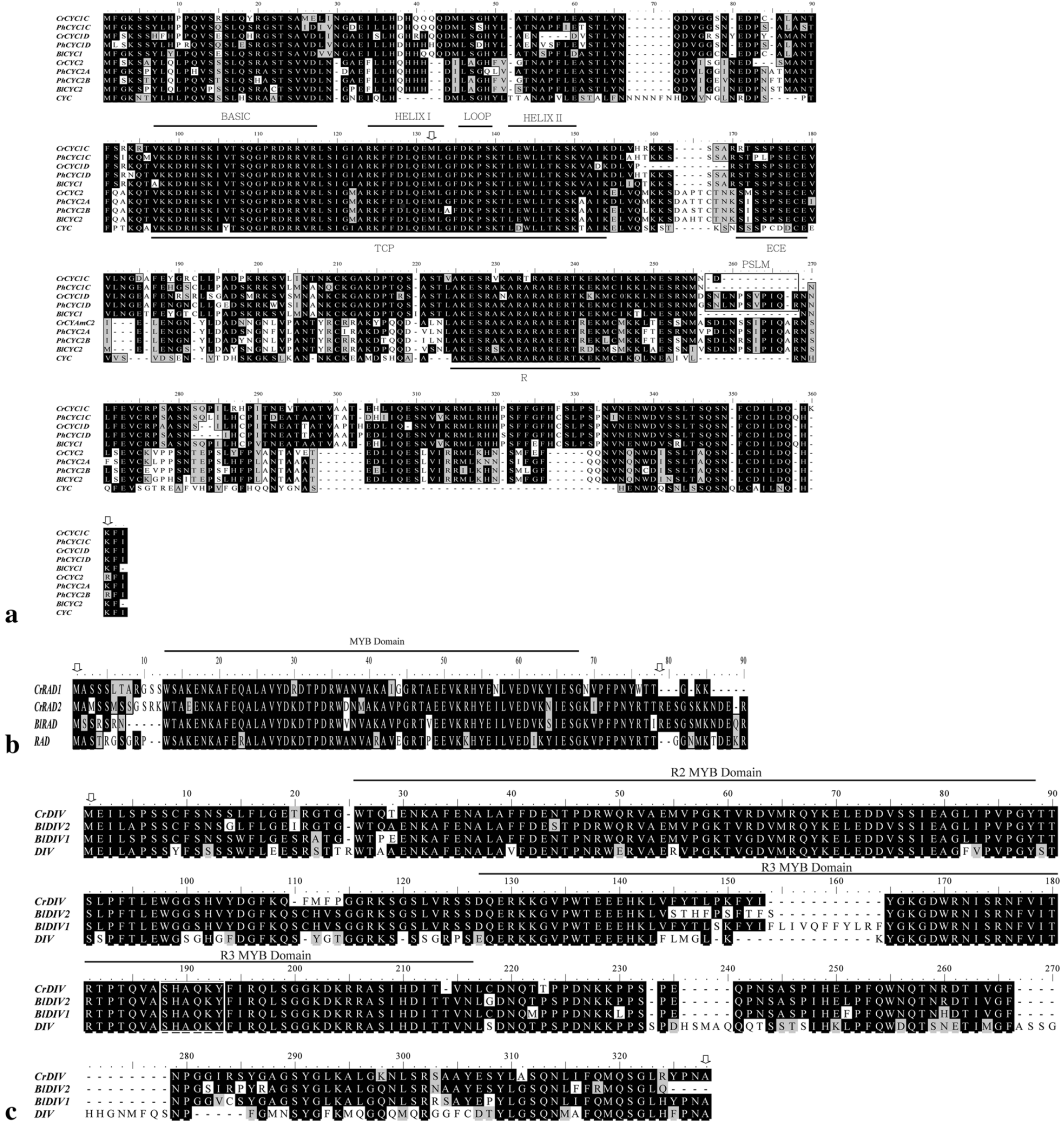
Alignments of protein sequences of *CrCYC*, *CrRAD* and *CrDIV* genes with homologs from *Antirrhinum majus* (*CYC*, *RAD* and *DIV*) and *Bournea leiophylla* (*BlCYC1*, *BlCYC2*, *BlRAD*, *BlDIV1* and *BlDIV2*). **a** Alignmentes of *CYC* homologs. TCP, ECE and R domain are outlined. Identical amino acids are in black and similar amino acids are in gray. **b** Alignments of *RAD* homologs. MYB domain are outlined. **c** Alignments of *DIV* homologs. Two MYB domains (R2 and R3) are outlined. A highly conserved SHAQKY motif in R3 MYB domain is identified and labelled in white box. Arrows indicate sequence region used in phylogeny analysis. Sequence region used in phylogenetic analysis coving almost all important domains in all three gene dataset except for *PlDIV* in *DIV* dataset

**Fig. 4:**
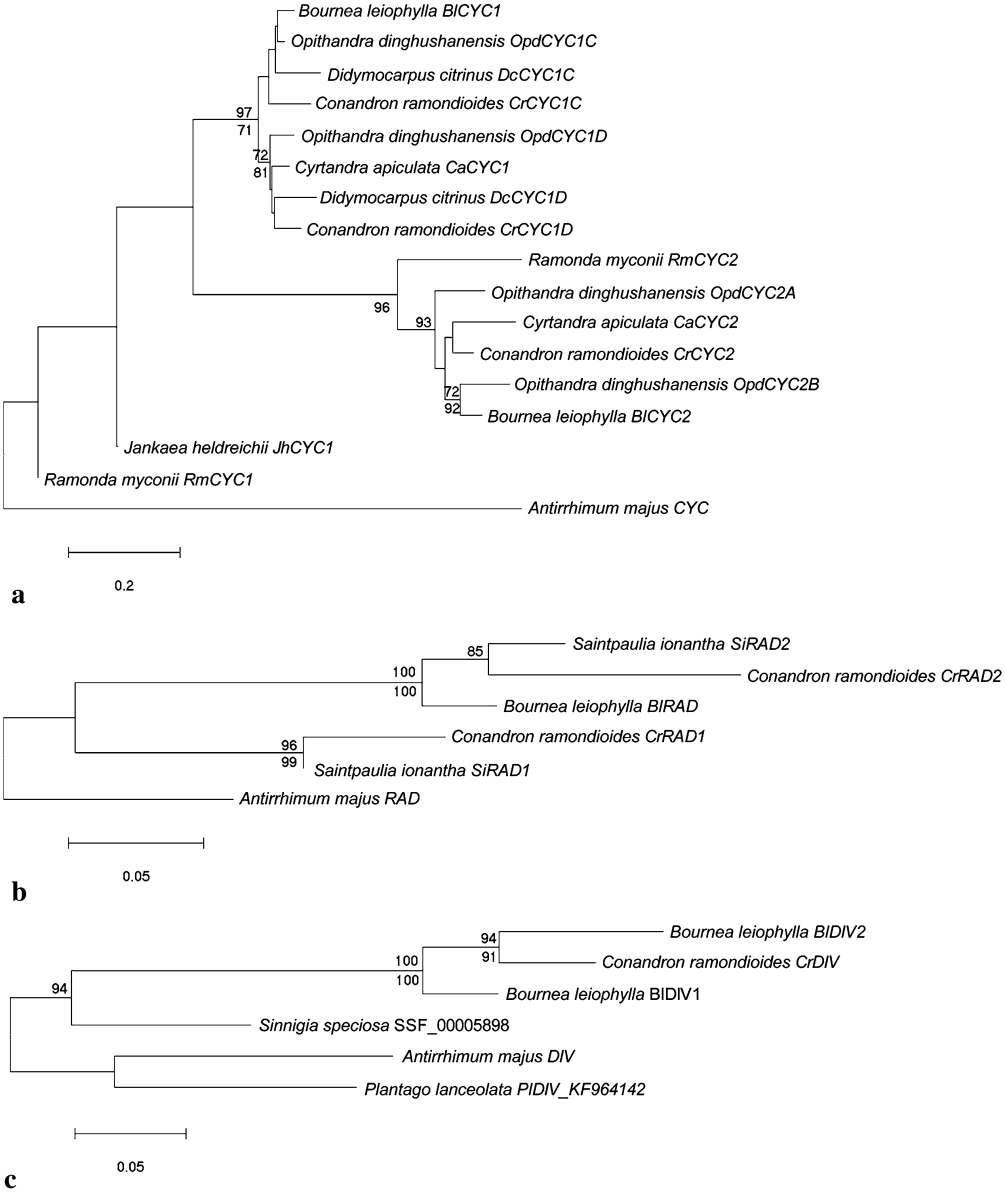
Neighbour-joining trees of *CYC*-like, *RAD*-like and *DIV*-like genes. Trees from **a** to **c** are reconstructed based on amino acid sequences. **a** *CYC* is from *A. majus*, others are *CYC*-like genes from Gesneriaceae species (see Additional file 2: Table S2). Trees show *CrCYCs* cluster into three groups with high support. **b** The tree shows *CrRADs* cluster into two distinct clade with high support. **c** Bootstrap values from NJ and ML are listed above and below branches respectively. Bootstrap values > 70 are shown. Detailed sequences information is listed in Additional file 2: Table S2

Among *CYC* clones, three out of 8 belonged to *CrCYC1C*, two belongs to *CrCYC1D* and three belongs to *CrCYC2*. For *CrRADs*, four clones belonged to *CrRAD1*, while the other four were *CrRAD2*. For *CrDIV*, all seven clones belonged to *CrDIV*. We believed our approach can effectively isolate all possible copies of each gene because we designed the primers in most conserved domain of each gene (see “Material and methods” section). Full-length cDNA of *CrCYC* genes, *CrRAD* genes and *CrDIV* were isolated from developing floral tissues and dissected tissues. The *CrCYC*, *CrRAD* and *CrDIV* sequences we isolated from *C. ramondioides* have been deposited in NCBI database (Accession number MH366524 to MH366529, detailed information see Additional file 2: Table S2). There are three *CYC* homologs, *CrCYC1C*, *CrCYC1D*, *CrCYC2* identified in *C. ramondioides* (Fig. 3a). Their full length amino acid sequences are 339, 338, and 335, respectively. According to phylogeny (Fig. 4a), we designated them as *CrCYC1C*, *CrCYC1D* and *CrCYC2*. Sequence analysis shows that *CrCYC1C*, *CrCYC1D* and *CrCYC2* are 26.1%, 17% and 23% identical to *Antirrhinum CYC*, respectively. When comparing the TCP domain, R domain, and ECE domains, *CrCYC1C*, *CrCYC1D,* and *CrCYC2* shared 92.6%, 90.2% and 92.6% amino acid sequence identity with *Antirrhinum CYC*, suggesting these genes are functionally related. When compared with *CYC*-like genes from available closely related Didymocarpoid Gesneriaceae species, *CrCYC1C* and *CrCYC1D* are 86% and 78.4% identical to *BlCYC1* of *Bournea leiophylla*, respectively, and *CrCYC2* is 88.1% identical to *BlCYC2*.

The sequence difference between these *CYC*-like genes are mainly located in the intervening regions of *CYC* domains mentioned above. Phylogenetic analysis shows that *CrCYC1C*, *CrCYC1D* (first isolated in this study) and *CrCYC2* formed three monophyletic clades (*GCYC1C*, *GCYC1D* and *GCYC2*) with other Gesneriaceae *CYC* homologs at amino acid level, confirming to previous phylogenetic trees (Fig. 4a).

Next, two *RAD* homologues isolated from *C. ramondioides* were *CrRAD1* and *CrDAD2*. They shared 76% and 68% amino acid identity with *RAD* from *A. majus*, respectively. Both *CrRAD1* and *CrDAD2* have one conserved 55aa-MYB-domain as *RAD* does (Fig. 3b). Phylogenetic analyses based on neighbor-joining method showed that *CrRAD1* and *CrRAD2* formed two distinct clades with high support (bootstrap/ML: 96/99 for *RAD1* clade; 100/100 for *RAD2* clade) (Fig. 4b). With *A. majus RAD* as outgroup, *CrRAD1* formed one monophyletic clade with Didymocarpoid Generiaceae species such as *RAD1* of *Saintpaulia ionantha* with high support, while *CrRAD2* formed another monophyletic clade with *Saintpaulia RAD2* and *RAD*-like gene of *B. leiophylla* according to nucleotide and amino acid NJ tree (Fig. 4b).

We only isolated one *DIV* homolog (*CrDIV*) from *C. ramondioides* which encode a protein of 296 amino acids. *CrDIV* is 54% identical to *DIV* from *A. majus* at amino acid level, and 90% and 89% identical to *BlDIV1* and *BlDIV2* from *B. leiophylla*. Conserved R2 and R3 domain and MYB-specific motif “SHAQKY” were found in *CrDIV* (Fig. 3c). Phylogenetic trees reconstructed from nucleotide and amino acid showed that *CrDIV* forms a monophyletic clade with *BlDIV1* (Fig. 4c).

### Tissue-specific expressions of *CrCYC*, *CrRAD and CrDIV*

**Fig. 5:**
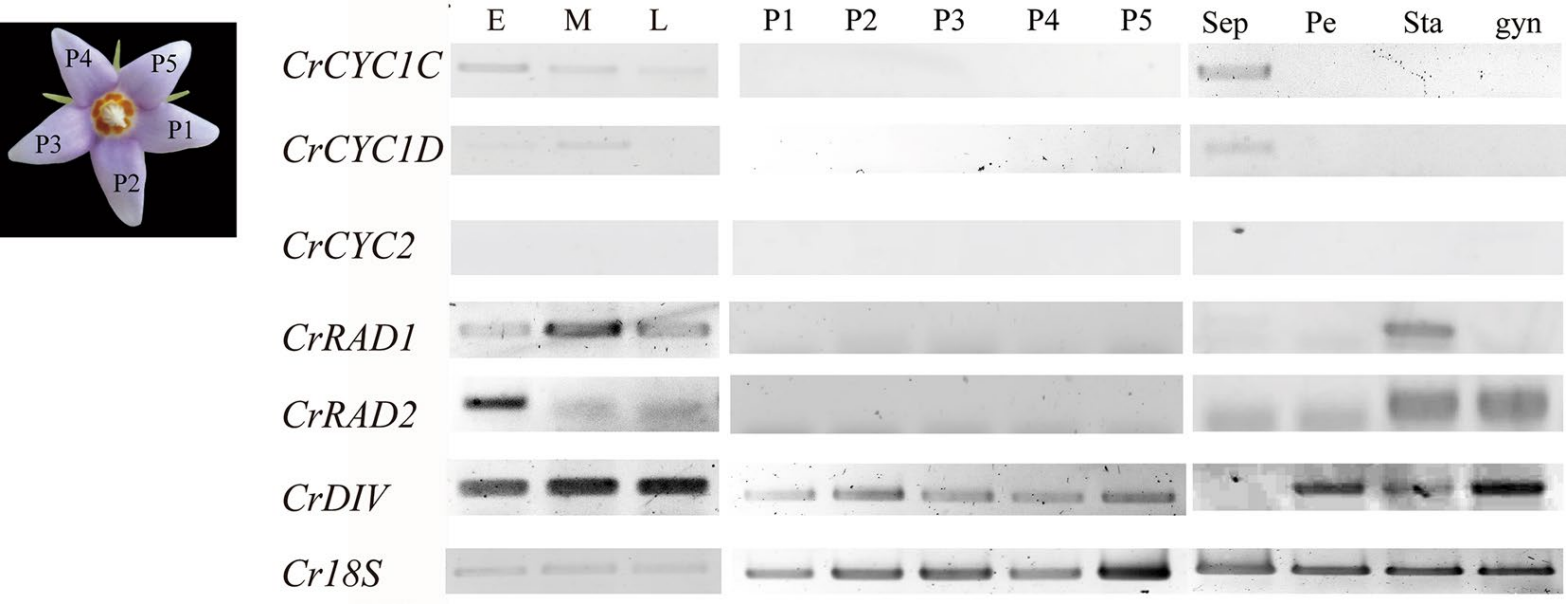
Gene-specific reverse transcriptase polymerase chain reaction (RT-PCR) analysis of *CrCYC*, *CrRAD* and *CrDIV* genes from *C. ramondioides* buds and dissected flower tissues. E, M, L represent three flower development stage defined in this study. E stands for early flower development stage; M stands for middle flower development stage; L stands for anthesis stage. P1 to P5 represent dissected petal from flower bud at early (E) flower developmental stage. Sep, Pe, Sta and gyn denote pooled sepals, pooled petals, pooled stamens and gynoecium dissected from early flower developmental stage. *CrCYC1C*, *CrCYC1D* and *CrCYC2* indicate expression of *CrCYC1C*, *CrCYC1D* and *CrCYC2*. *CrRAD1* and *CrRAD2* indicate expression of *CrRAD1* and *CrRAD2*. *CrDIV* indicates expression of *CrDIV*. *18S* is included as a positive control. *CrCYC1C*, *CrCYC1D*, *CrRAD1*, *CrRAD2* and *CrDIV* are detected through flower development stages, only *CrCYC2* is restricted through flower development stages. *CrDIV* is detected in all petals, whereas *CrCYC1C*, *CrCYC1D*, *CrCYC2*, *CrRAD1* and *CrRAD2* are restricted in petals. *CrCYC1C* and *CrCYC2* were detected in pooled sepal tissue

Transcripts of *CrCYC1C* and *CrCYC1D* were first detected in the early developmental stage (E) of the floral bud and then the expression of *CrCYC1C* and *CrCYC1D* were gradually reduced to almost invisible from middle (M) to late stage (L) of entire floral buds (Fig. 5). To further confirm organ expression pattern of *CrCYC1C* and *CrCYC1D*, pooled tissues including petals, gynoecium, stamens and sepals dissected from early development stage were used. Surprisingly, transcripts of *CrCYC1C* and *CrCYC1D* were both only detected in sepals (Fig. 5). This unique expression pattern of *CrCYC1C* and *CrCYC1D* in *C. ramondioides* seems to correlate with the residual zygomorphy found in sepal whorl (see Fig. 2a). As to *CrCYC2*, its expression was undetectable throughout all flower developmental stages and dissected organs. In summary, our RT-PCR results showed that no *CrCYCs* transcripts were detectable at petal and stamen whorl.

As to *CrRADs*, *CrRAD1* expression can be detected throughout all flower developmental stages but *CrRAD2* could only be detected in the early floral development stage (Fig. 5). Their organ expression pattern revealed that both *CrRAD1* and *CrRAD2* expressed in stamens, while *CrRAD2* also expressed in gynoecium (Fig. 5). The *CrDIV* was continuously expressed throughout all flower developmental stages. Specifically, *CrDIV* expressed in petals, stamens and gynoecium but not in sepals (Fig. 5).

## Discussion

### Reversal to actinomorphy correlate to diverse *CYC* expression shifts in Gesneriaceae and other eudicot lineages

Our SEM pictures showed that all five petals of *C. ramondioides* seems to grow in consistent rate of enlargement ever since petal primordia initiation stages (Fig. 2). Similarly, this was also observed in the stamen whorl. Development of actinomorphy in *C. ramondioides* therefore resembles to that in *cyc*/*dich* mutant of *A. majus* in which all petal primordia maintain equal growth rate along flower developmental stages and all stamens are fully developed (Luo et al. 1996).

From RT-PCR results, *CrCYC*s and *CrRAD*s have no sign of expression in petal whorl (Fig. 5). This suggests *C. ramondioides* is a ventralized actinomorphy. In Gesneriaceae, ventralized actinomorphy has been reported in the peloric cultivar of *Sinningia speciosa* where *CYC* is not expressed in petal whorl because a deletion in *CYC* coding region (Hsu et al. 2015, 2017; Wang et al. 2015). Loss of *CYC* expression in late petal developmental stage but not in early stage can also contribute to actinomorphic reversal in *B. leiophylla* (Zhou et al. 2008). On the other hand, dorsalized actinomorphy due to ubiquitous *CYC* expression in entire petal whorl has been reported in *T. scopulorum* and occasionally in *Petrocosmea* hybrids (Pang et al. 2010; Yang et al. 2015). Other than Gesneriaceae, examples of dorsalized actinomorphy have been reported in *Cardia purpurea* (Leguminosae), in *Aquilegia alpina* (Ranunculaceae), and in certain Malpighiaceae lineages (Citerne et al. 2006; Zhang et al. 2010, 2012, 2013; Jabbour et al. 2014). While ventralized actinomorphy where the loss of *CYC* expression has been documented in *Tradescantia* (Commelinaceae), in *Nigella damascene* (Ranunculaceae), in *Plantago lanceolata* (Plantaginaceae), in Malpighiaceae lineages and in *Arabidopsis* (Cubas et al. 2001; Reardon et al. 2009; Preston et al. 2011; Preston and Hileman 2012; Zhang et al. 2013; Jabbour et al. 2014).

Although we did not detect any *CrCYC1C* and *CrCYC1D* expression in petal and stamen whorls, we did find them distinctively expressed in sepals. Sepal specific expression of *CYC* has been inferred the ancestral state among Ranunculales including both actinomorphic Papaveraceae species and zygomorphic Fumariaceae species, the basal most core Eudicot lineage (Damerval et al. 2007). Thus, the shifts of *CrCYC1C*/*1D* back to ancestral expression in sepals and cease of expression in petals/stamens seem to associate with the actinomorphic reversal in *C. ramondioides.* Indeed, the recruitment of *CYC* expression (ECE clade) in sepal was evolved earlier than expression in dorsal-specific manner (Preston and Hileman 2009). It would be interesting to examine whether all those reversals to actinomorphy species (Wang et al. 2010; Clark et al. 2011) in Geseneriaceae also have their *CYC* expression return to ancestral state of sepal expression.

### Duplications of *CYC* may associate to expression shift and flower shape variation

Our phylogenetic analysis revealed that the three *CrCYCs* we isolated (*CrCYC1C*, *CrCYC1D* and *CrCYC2*) were resulted from at least two duplication events among Gesneriaceae species, congruent to previous findings (Möller et al. 1999; Citerne et al. 2000; Wang et al. 2004; Du and Wang 2008; Song et al. 2009; Pang et al. 2010; Yang et al. 2015). Although these *CrCYCs* have lost their expression in petal and stamen whorls, which is correlate to the reversal to actinomorphy in *C. ramondioides*, their coding sequences contain no frame shift or nonsense mutations. This implies these *CrCYCs* may still function yet the regulation controls on cis-elements in their promoter regions could have been mutated to become mis-expression (i.e. sepal only expression). It would be interesting to test this hypothesis by ectopically express these *CrCYCs* separately in *Arabidopsis* and compare their phenotypic effects.

In snapdragon, *CYC* evolved as major effect copy (higher and broader dorsal-specific expression) and its duplicates *DICH* as helper function (Luo et al. 1996, 1999). Similarly, *CYC* tend to duplicate in most Angiosperm lineages with either both copies retain similar expression pattern (major/helper) or expressions become diversified thus under different selection pressures (Ree et al. 2004; Chapman et al. 2008; Bello et al. 2017). In *C. ramondioides*, *CrCYC1C* and *CrCYC1D* both have sepal only expression but expression level of *CrCYC1C* is higher than *CrCYC1D*. This is similar to the case of *CYC* and *DICH* in snapdragon. Duplication could allow one copy to maintain the essential function but the other to evolve into novel or modified function. *CYC* duplications may therefore link to the evolution of diverse floral shape in angiosperms, although yet to be determined. There are reports, however, indicating shifts of expression between *CYC* paralogs correlate to floral symmetry transitions and/or flower shape variations (Bartlett and Specht 2011; Zhang et al. 2012; Jabbour et al. 2014).

### Specific expression patterns of *CrRADs* and *CrDIV* may suggest loss of antagonistic expression pattern between *CrRADs* and *CrDIV* following loss of *CYC* expression in *C. ramondioides*

In *cyc*/*dich* double mutant *A. majus*, *RAD* does not express in dorsal region, thus, allowing ventral region restricted *DIV* to spread to whole flower (Corley et al. 2005). In *B. leiophylla*, once dorsal region restricted *BlCYC1* and *BlRAD* are downregulated, *BlDIV* spread to corolla and stamen whorl (Zhou et al. 2008). To sum up, *RAD*-like genes and *DIV*-like genes seem have antagonistic function in *A. majus* and *B. leiophylla*. However, expression patterns of *CrRADs* and *CrDIV* are different from those gene expression pattern in *A. majus* and *B. leiophylla*. In *C. ramondioides*, both *CrRADs* and *CrDIV* expressed in stamens (including *CrRAD1*, *CrRAD2* and *CrDIV*) and gynoecium (including *CrRAD2* and *CrDIV*) at the same development stage (Fig. 5). Comparing expression patterns of *RAD*-like and *DIV*-like genes among these three species, antagonistic function seem have lost in *C. ramondioides*. Based on expression patterns from these three species, we postulate that loss of expression of upstream gene (e.g. *CYC* and its homologue) may provide opportunity for its downstream gene (e.g. *RAD* and its homologue, *DIV* and its homologue) releasing from genetic constraint. To conclude, antagonistic expression pattern between *RAD*-like and *DIV*-like genes was maintained in *B. leiophylla*, which resembling to *CYC*-mediated regulatory pathway in *A. majus*. However, this antagonistic expression pattern was lost in *C. ramondioides*. Since the *RAD*-like and *DIV*-like genes were rarely studied in Gesneriaceae, it would be interesting to examine whether the maintaining or loss of antagonistic function between *RAD*-like and *DIV*-like genes is a common pattern or not in Gesneriaceae.

### Reversal to actinomorphy may help to attract general pollinator visiting *C. ramondioides*

In *C. ramondioides*, reversal to actinomorphy coupling with very short corolla tube at anthesis may facilitate generalist (e.g. bees, small beetles) visitation because they can obtain pollen from any direction (Fig. 1). Similarly, it has been postulated that reversal to actinomorphy in *R. myconii* could allow visits from a wider range of pollinators in alpine extreme habitats (Cronk and Möller 1997). In alpine or harsh conditions, plants may suffer low pollinator visiting. If certain zygomorphic species still rely on their specific pollinator visiting in harsh condition, their reproductive success may be low. But if species which can reverse to actinomorphy by opening the corolla, such as *C. ramondioides* and *R. myconii*, they could have pollinator shifts to a variety of pollinators to increase visiting rate. Although *C. ramondioides* is not distributed in alpine environment like *R. myconii*, its deep-forest dense shape habitat may discourage insect pollinator visiting (Peat and Goulson 2005). Reversal to actinomorphy to attract more general pollinators in pollinator scarce habitats may actually compensate for maintaining the reproductive success in *C. ramondioides*. To support this idea, detailed pollination experiment and pollinator observation in the field are necessary in the future.

## Additional files

**Additional file 1: Table S1.** Development procedure of C. ramondioides flower.

**Additional file 2: Table S2.** Sequences used to reconstruct CYC, RAD and DIV tree.

**Additional file 3: Figure S1.** Biological repeats of gene-specific reverse transcriptase polymerase chain reaction (RT-PCR) analysis of *CrCYC*, *CrRAD* and *CrDIV* genes from *C. ramondioides* buds and dissected flower tissues. (a), (b) represent two biological repeats respectively. All abbreviations correspond to descriptions in Fig. 4.

## Abbreviations

Sep: pooled sepals
Pe: pooled petals
Sta: pooled stamens
gyn: gynoecium
