# Supplementary material for: Expression shifts of floral symmetry genes correlate to flower actinomorphy in East Asia endemic Conandron ramondioides (Gesneriaceae)
Source: Bot Stud. 2018 Oct 29;59:24. doi: 10.1186/s40529-018-0242-x (PMC6206312; doi:10.1186/s40529-018-0242-x)
Supplement: Supplementary file 3 — Additional file 3: Figure S1. Biological repeats of gene-specific reverse transcriptase polymerase chain reaction (RT-PCR) analysis of CrCYC, CrRAD and CrDIV genes from C. ramondioides buds and dissected flower tissues. (a), (b) represent two biological repeats respectively. All abbreviations correspond to descriptions in Fig. 4. [file 40529_2018_242_MOESM3_ESM.docx]

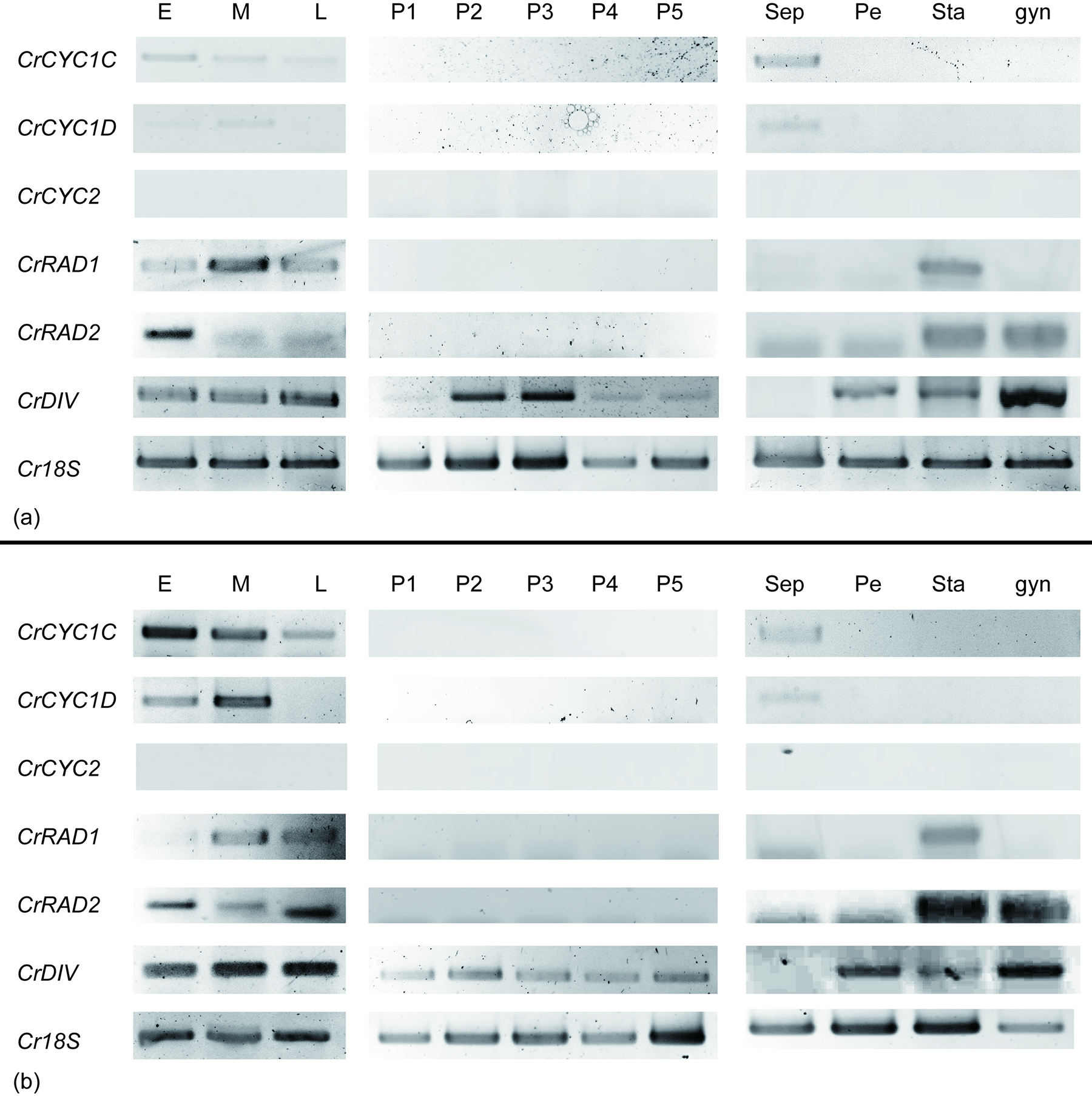


Figure S1 Biological repeats of gene-specific reverse transcriptase polymerase chain reaction (RT-PCR) analysis of *CrCYC*, *CrRAD* and *CrDIV* genes from *C. ramondioides* buds and dissected flower tissues. (a), (b) represent two biological repeats respectively. All abbreviations correspond to descriptions in Fig. 5.
